# Neuroprotective effect of naringin against cerebellar changes in Alzheimer’s disease through modulation of autophagy, oxidative stress and tau expression: An experimental study

**DOI:** 10.3389/fnana.2022.1012422

**Published:** 2022-10-14

**Authors:** Hend M. Hassan, Mohamed R. Elnagar, Eman Abdelrazik, Mohamed R. Mahdi, Eman Hamza, Eman M. Elattar, Eman Mohamed ElNashar, Mansour Abdullah Alghamdi, Zainah Al-Qahtani, Khulood Mohammed Al-Khater, Rashid A. Aldahhan, Mamdouh ELdesoqui

**Affiliations:** ^1^Department of Human Anatomy and Embryology, Faculty of Medicine, Mansoura University, Mansoura, Egypt; ^2^Department of Pharmacology and Toxicology, Faculty of Pharmacy, Al-Azhar University, Cairo, Egypt; ^3^Department of Pharmacology, College of Pharmacy, The Islamic University, Najaf, Iraq; ^4^Department of Forensic Medicine and Clinical Toxicology, Faculty of Medicine, Mansoura University, Mansoura, Egypt; ^5^Department of Medical Biochemistry and Molecular Biology, Faculty of Medicine, Mansoura University, Mansoura, Egypt; ^6^Department of Medical Biochemistry and Molecular Biology, Faculty of Medicine, Horus University, Damietta, Egypt; ^7^Department of Pharmacognosy, Faculty of Pharmacy, Mansoura University, Mansoura, Egypt; ^8^Department of Anatomy, College of Medicine, King Khalid University, Abha, Saudi Arabia; ^9^Department of Histology and Cell Biology, Faculty of Medicine, Benha University, Banha, Egypt; ^10^Genomics and Personalized Medicine Unit, College of Medicine, King Khalid University, Abha, Saudi Arabia; ^11^Neurology Section, Department of Internal Medicine, College of Medicine, King Khalid University, Abha, Saudi Arabia; ^12^Department of Anatomy, College of Medicine, Imam Abdulrahman Bin Faisal University, Dammam, Saudi Arabia; ^13^Department of Basic Medical Sciences, College of Medicine, AlMaarefa University, Riyadh, Saudi Arabia

**Keywords:** naringin, autophagy, cerebellum, Alzheimer’s disease, oxidative stress, aluminum chloride

## Abstract

Alzheimer’s disease (AD) is a neurodegenerative disorder characterized by gradual cognitive decline. Strong antioxidants that inhibit free radicals, such as polyphenols, reduce the likelihood of developing oxidative stress-related degenerative diseases such as AD. Naringin, a flavonoid found in citrus fruit shown to be neuroprotective, reduce oxidative damage and minimize histopathological changes caused by ischemic reperfusion, enhance the long-term memory in AD animal models. This work aimed to comprehend the role of naringin in the defense of the cerebellum against aluminum chloride (AlCl_3_)-induced AD in rats by investigating the behavioral, neurochemical, immunohistochemical, and molecular mechanisms that underpin its possible neuroprotective effects. Twenty-four adult albino rats were divided into four groups (*n* = 6/group): (i) Control (C) received saline per oral (p.o.), (ii) Naringin(*N*)-received naringin (100 mg/kg/d) p.o, (iii) AlCl_3_-recived AlCl_3_ (100 mg/kg/d) p.o and (iv) AlCl_3_ + Naringin (AlCl_3_ + *N*) received both AlCl_3_ and naringin p.o for 21 days. Behavioral tests showed an increase in the time to reach the platform in Morris water maze, indicating memory impairment in the AlCl_3_-treated group, but co-administration of naringin showed significant improvement. The Rotarod test demonstrated a decrease in muscle coordination in the AlCl_3_-treated group, while it was improved in the AlCl_3_ + *N* group. Neurochemical analysis of the hippocampus and cerebellum revealed that AlCl_3_ significantly increased lipid peroxidation and oxidative stress and decreased levels of reduced glutathione. Administration of naringin ameliorated these neurochemical changes *via* its antioxidant properties. Cerebellar immunohistochemical expression for microtubule assembly (tau protein) and oxidative stress (iNOS) increased in A1C1_3_-treated group. On the other hand, the expression of the autophagic marker (LC3) in the cerebellum showed a marked decline in AlCl_3_-treated group. Western blot analysis confirmed the cerebellar immunohistochemical findings. Collectively, these findings suggested that naringin could contribute to the combat of oxidative and autophagic stress in the cerebellum of AlCl_3_-induced AD.

## Introduction

Alzheimer’s disease (AD) is the most prevalent neurodegenerative disease that causes memory loss and progressive neurocognitive deterioration in the elderly (Babri et al., 2014). A key risk factor for various age-related neurodegenerative diseases, such as AD, is aluminum (Al) (Kumar et al., 2011). It has been widely used in the industry, and it is currently added to a large number of products available to everyone, including drinking water, many processed foods, infant formulae, cosmetics, toothpaste, antiperspirants, and various medical preparations and medicines (Bondy, 2016).

AlCl_3_ is a strong neurotoxin that has been linked to the neuropathogenesis of AD (Borai et al., 2017). It is reported to be involved in the etiology of AD as it can easily cross the blood brain barrier (Lakshmi et al., 2015; Borai et al., 2017). Al ions are able to interact with different proteins inducing misfolding and aggregation which are key pathophysiological mechanisms in AD (Colomina and Peris-Sampedro, 2017).

The hippocampus, cerebral cortex, cerebellum, corpus callosum, amygdala, thalamus, and corpus callosum are among the areas of the brain that exhibit slow and progressive neurodegeneration in AD (Akbarpour et al., 2015). Cerebellum has long been known for its role in motor control, but more recently, it has also been regarded important for higher-order cognitive, emotional, and even social processing (Schmahmann, 2019). It exhibits neuropathology in AD which includes structural and functional abnormalities that match the geography of neurodegeneration seen in the cerebral hemispheres (Jacobs et al., 2018).

Motor and cognitive abilities deteriorate with age in both humans and animals, which may be related to a greater vulnerability to the cumulative effects of oxidative stress and inflammation (Zhang et al., 2013). There are evidences that brain tissue in AD patients is exposed to oxidative stress (Gella and Durany, 2009). The most significant feature is that oxidative stress appears to be a primary progenitor of the disease (Bonda et al., 2010).

Polyphenols are strong antioxidants that inhibit developing oxidative stress-related degenerative diseases such as AD (Basli et al., 2012). Their ability to improve neurological health is mediated by several mechanisms, including their interaction with neuronal and glial signaling pathways, reduction in neurotoxins-mediated neuronal damage and loss or neuroinflammation, decrease in reactive oxygen species (ROS) production, and decrease in the accumulation of neuropathological markers like amyloid-β (Aβ) and tau protein (Bensalem et al., 2015).

One of the most significant flavonoids that can be extracted from citrus fruits is naringin (NAR). Due to its potent antioxidant, anti-inflammatory, antiapoptotic, anti-ulcer, anti-osteoporosis, and anticancer properties, it has gained interest (Chen et al., 2016). Previous studies have shown that naringin therapy can reduce oxidative damage and minimize histopathological changes caused by ischemic reperfusion in the brain, striatum, and hippocampus (Cao et al., 2021). Furthermore, previous research has shown that it can enhance long-term memory and act as a neuroprotective agent in transgenic AD mice (Wang et al., 2013).

Tau protein, which plays an essential role in microtubule construction and stabilization, supports appropriate neuronal activity. Aberrant tau protein phosphorylation has been associated with AD progression, as well as its ability to cause cytotoxicity when produced in cultured cell *in vitro* and animal models (Kolarova et al., 2012).

AD is also associated with autophagic stress in which the rate of autophagosome production is greater than the rate of breakdown in response to protein or organelle damage (Farrag et al., 2021). Furthermore, strong autophagic activity can eliminate damaged mitochondria, so indirectly reducing the amount of ROS production and activation of the inflammasome (Zhao et al., 2019).

Since the neurochemical, immunohistochemical, and molecular effects of naringin on cerebellar neurotoxicity in AlCl_3_-induced AD have not been investigated yet, this study aims to explore the role of naringin in ameliorating cerebellar changes of AlCl_3_ rat model of AD.

## Materials and methods

### Ethics approval

Following NIH and EU norms for animal care, this study received Institutional Research Board (IRB) approval from Mansoura Faculty of Medicine (Code number: R.21.03.1280). At the Medical Experimental Research Center (MERC), where the experiment was carried out, the rats were housed under veterinary care. The number of animals used and animal discomfort were minimized as far as possible.

### Animals

Twenty-four adult (3 months old) male albino rats of average weight of about 200–250 g were used. They were given unrestricted access to food and drink during the regular day-night cycle (12–12 h).

### Chemicals

AlCl_3_ and naringin (N1376) were obtained in powder form from Sigma-Aldrich.

### Experimental design

After 2 weeks of acclimatization, the rats were divided into four groups (*n* = 6/group): (i) Control (C) received saline per oral (p.o.), (ii) Naringin(*N*)-received naringin (100 mg/kg/d) p.o (Meng et al., 2021), (iii) AlCl_3_-recived AlCl_3_ (100 mg/kg/d) p.o (Lakshmi et al., 2015), and (iv) AlCl_3_ + Naringin (AlCl_3_ + *N*) received both AlCl_3_ and naringin p.o for 21 days. At the end of experiment, body weight was measured and Rotarod and Morris water maze tests were conducted to assess motor and learning abilities, respectively. On sacrifice, the hippocampus was dissected out to confirm neuronal degeneration. The cerebellum also was excised, neurochemical estimation of oxidative stress markers was performed, Histopathological and immunohistochemical evaluation of autophagy, Tau and oxidative stress were done and Western blotting was performed.

### Body weight measurement

At the end of the experiment, rats were weighed to detect if there were significant differences in their weight after exposure to naringin and AlCl_3_.

### Behavioral assessment

#### Rotarod test for assessment of motor ability and muscle coordination

Two initial training trials of 5 min each were administered to each rat to maintain posture on the rotarod, which had a diameter of 3 cm and rotated at a constant 20 rev/min. These trials were spaced roughly 10 min apart. After training trials, a trial of 2 min was done for each rat where time spent on the rotarod (grip period) was noted (Ataie et al., 2010).

#### Morris water maze test for the evaluation of learning capacity and spatial memory

Morris water maze is composed of learning (acquisition) and retention phases. The Maze consists of a large circular pool (150 cm in diameter, 45 cm in height, filled to a depth of 30 cm with water at 28 ± 1^°^C) divided into four equal quadrants. For learning of memory, a circular platform (4.5 cm diameter) was placed in one quadrant of the pool 1 cm below the water level. Each rat was subjected to four consecutive trials with a gap of 5 min. The rat was gently placed in the water of the pool between quadrants facing the wall of the pool. Each rat was then allowed 120 s to locate the platform. If the animal failed to reach the platform within 120 s, it was guided to it and allowed to remain there for 20 s. The time taken by each rat in each group to reach the platform (escape latency) was calculated using a stopwatch. For retention of memory (4 h after the last learning session), the platform was removed and the entry latency to the platform quadrant was detected (Laskowitz et al., 2017).

### Sample preparation

One day after the final dose of the treatment, the rats were anesthetized with 300 mg/kg of intraperitoneal chloral hydrate. The hippocampus and cerebellum were carefully removed. A portion was processed to create the paraffin blocks. Cut sections (4 μm thick) were obtained. Hematoxylin and eosin staining (H & E) was performed for both tissues. Cerebellar sections were also immunostained for the expression of LC3, tau protein, and iNOS. Another cerebellar portion was then preserved in RNA later (for RNA and protein stabilization) (Thermo Fisher Scientific, Waltham, MA, USA) at (−20^°^C) before being stored at (−80^°^C) until extraction of protein and subsequent Western blot analysis. Fresh portions of the hippocampus and cerebellum were used to create homogenates for the evaluation of oxidative stress markers.

### Neurochemical evaluation of oxidative stress markers malondialdehyde, nitric oxide, and reduced glutathione

The tissues of the hippocampus and cerebellum were rinsed with ice and thoroughly cleaned. They were then weighed in an analytical balance after being softly wiped between filter paper folds. A polytron homogenizer was used to prepare 10% of the homogenate at 4^°^C in 0.05 M phosphate buffer (pH 7). The homogenate was centrifuged at 10,000 rpm for 20 min to separate unbroken cells, mitochondria, erythrocyte nuclei and cell debris. According to the directions in the handbook, the supernatant was aliquoted and kept at −80^°^C for further analysis of MDA (Barros et al., 2017), NO (Bhidwaria and Ashwlayan, 2017) and GSH (Pires et al., 2014). To estimate their levels, commercial colorimetric kits were used from the Biodiagnostic Company (Cairo, Egypt).

### Immunohistochemical detection of LC3 (autophagy marker), iNOS (oxidative stress marker) and tau protein (for the construction and stabilization)

Immunohistochemical localization of LC3, iNOS, and tau protein was performed as previously described. Briefly, 0.03% H_2_O_2_ was used to block endogenous peroxidases. The antigens were heated in a microwave for 20 min with sodium citrate buffer (pH 6), and then buffered saline containing 5% bovine serum albumin. Subsequently, sections were incubated with a primary antibody against tau (1:100, ab92676), LC3 (1:300, ab48394), and iNOS (1:2,000, ab283655) for an entire night at 4^°^C. Following the manufacturer’s instructions, the avidin-biotinylated peroxidase complex (ABC-kit) and DAB substrate (ab64238) were used to detect the response. Finally, hematoxylin was used as a counterstain, and sections were dehydrated in ascending grades of alcohols, cleared in xylene, and mounted (Chen et al., 2010).

### Measurement of hippocampal pyramidal cell count

To estimate the number of pyramidal cells in the cornu amonnis region 1 (CA1), five randomly spaced H & E-stained hippocampal sections for each rat in each group were examined. Five photomicrographs from each section were used. Then the count of pyramidal neurons was detected in the calibrated area (0.43 mm^2^) using the Image J program (Version 1.48) and the cell counter plugin. The analysis was done at a magnification of × 200 (Mavroudis et al., 2019).

### Measurement of the length of Purkinje cell dendrites

Five randomly spaced cerebellar sections stained with H & E for each rat in each group were examined. Five photomicrographs from each section were used. The length of the Purkinje cell dendrites (μm) was detected in the calibrated area (area: 0.071 mm^2^) using the Image J program (Version 1.48). The analysis was done at a magnification of × 400 (Chen H. et al., 2019).

### Measurement of % area of positive LC3, tau, and iNOS immunoreaction in cerebellar tissue

Five randomly spaced sections for each rat in each group were examined. Five photomicrographs from each section were used. The area fraction of immunological expression was calculated using a 40 × objective (area: 0.071 mm^2^). Immune-positive reaction was analyzed using the Image-j computerized image analysis system (version1.48). Using the color deconvolution plugin and H-DAB vector, three distinct colored images; green, brown, and blue were produced. By calculating area fraction, the DAB pictures (brown in color) were calibrated. The threshold was adjusted for more precision (Helmy et al., 2022).

### Western blot analysis for the detection of the expression of p-Tau, Tau, iNO, and LC3-II/I proteins

The expression of p-Tau, Tau, iNOs, and LC3-II/I proteins in the cerebellum was determined using Western blot procedure (Elnagar et al., 2017). Briefly, tissues were homogenized in 250μl pre-cold lysis buffer pH 7.4; 10 mM Tris-Base, 100 mM NaCl, 20 mM Ethylene EGTA, 25 mM EDTA, 2% Triton X-100, and 1:350 protease and phosphatase inhibitor cocktail (Sigma). The tissue homogenates were immediately centrifuged at 12,000 rpm for 15 min. Total proteins were determined using the Pierce 660 nm assay (Thermo Scientific, Rockford, IL). Then, equal amounts (25 μg) of protein were mixed with the loading buffer contains Tris-HCl, dithiothreitol (DTT), sodium dodecyl sulfate (SDS), glycerol, and Bromophenol blue. Protein samples were boiled for 5 min at 95^°^C, allowed to cool on ice for 5 min, and separated by electrophoresis (Cleaver Scientific Ltd., UK). The proteins on the gel were then electroblotted onto PVDF membranes for 35 min using a Trans-Blot^®^ SD semi-dry transfer cell (Biorad). PVDF membranes were blocked with 5% dry milk (Biorad) in Tris buffered saline supplemented with Tween-20 (TBS-T). The membranes were washed and incubated with antibodies against p-Tau, Tau, iNO, LC3-II/I proteins (1:1,000, Cell Signaling Technology) and β-actin (1:3,000, Sigma) proteins for 13–15 h at 4^°^C. The membrane blots were incubated with secondary antibodies for 2 h at RT before they visualized with ECL chemiluminescence reagents (Perkin Elmer, USA) for 2 min on the Biorad Chemi-Doc imager, and finally the band intensities were analyzed with ImageLab^§^ software (Biorad) with normalization to β-actin.

### Statistical analysis

Version 26.0 of IBM SPSS for Windows was used to analyze the data. The Shapiro test of normality results showed that the data were normally distributed. They were labeled as mean ± *SD*. The significance was assessed at the (0.05) level. The means of the 4 study groups were compared using the one-way ANOVA test and the *Post Hoc* Games-Howell test was used to identify pairwise comparisons.

## Results

### Body weight assessment results

At the end of the experiment, there were significant differences in body weight among the four studied groups (*p* < 0.001, ANOVA test). There was no significant difference between C and *N* groups (*p* = 0.9). Also, the difference between C and AlCl_3_ + *N* groups was insignificant (*p* = 0.1). AlCl_3_ administration resulted in a significant drop in the body weight as compared to both C and N groups (*p* < 0.001). Co-administration of naringin with AlCl_3_ resulted in a significant increase in body weight as compared to AlCl_3_ group (*p* = 0.006) (Table 1).

**TABLE 1:** Effects of AlCl_3_ and naringin administration on body weight.

	C	*N*	AlCl3	AlCl_3_ + N	*P*-value
Body weight (gm)	223.3 ± 10.8	227.5 ± 14.4^#^	163.3 ± 19.7*	208.7 ± 9.4^$^	*p* < 0.001**

*n* = 6 in each group. Data were presented as mean ± SD.

**Indicates significance among the studied groups indicated by ANOVA test. The intergroup variation was conducted by Games-Howell post-hoc test.

*p < 0.001 compared with C group.

^#^*p* < 0.001 compared with AlCl_3._

^$^*p* = 0.006 compared with AlCl_3_.

### Behavioral assessment results

**FIGURE 1:**
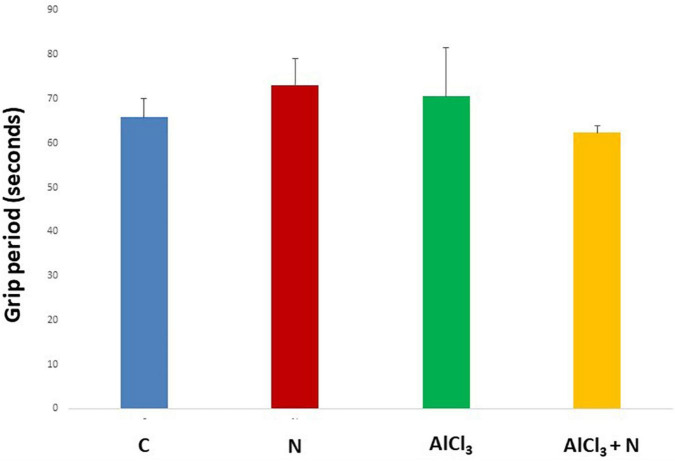
Grip period in the Rotarod test during initial training periods in the studied groups. *n* = 6 in each group. Data were prescribed as mean ± *SD*. No significant differences were detected between any groups (*p* > 0.05, Games-Howell *post-hoc* test).

**FIGURE 2:**
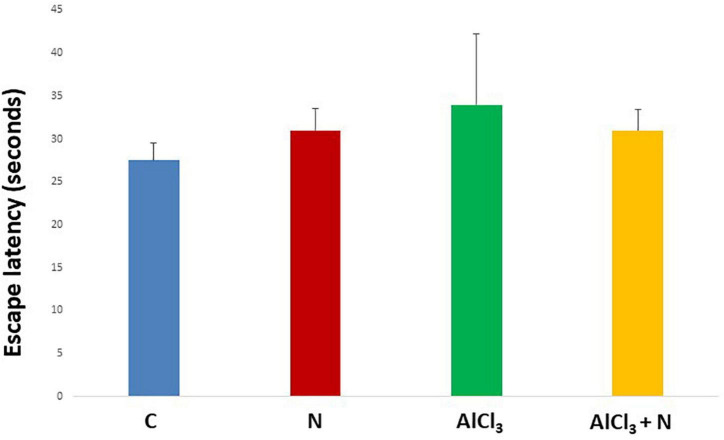
Escape latency in the Morris water maze test during initial training periods in the studied groups. *n* = 6 in each group. Data were prescribed as mean ± *SD*. No significant differences were detected between any groups (*p* > 0.05, Games-Howell *post-hoc* test).

**FIGURE 3:**
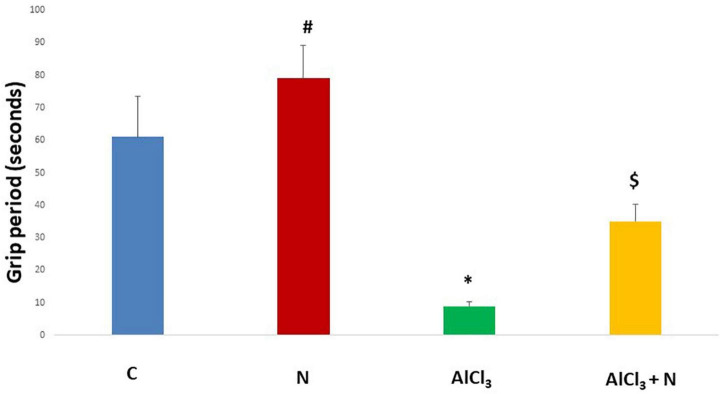
Grip period in the Rotarod test in the studied groups. *n* = 6 in each group. Data were prescribed as mean ± *SD*. The intergroup variation was conducted by Games-Howell *post-hoc* test. *p < 0.001 compared with C group. ^#^*p* < 0.001 compared with AlCl_3._^$^*p* = 0.01 compared with AlCl_3_.

**FIGURE 4:**
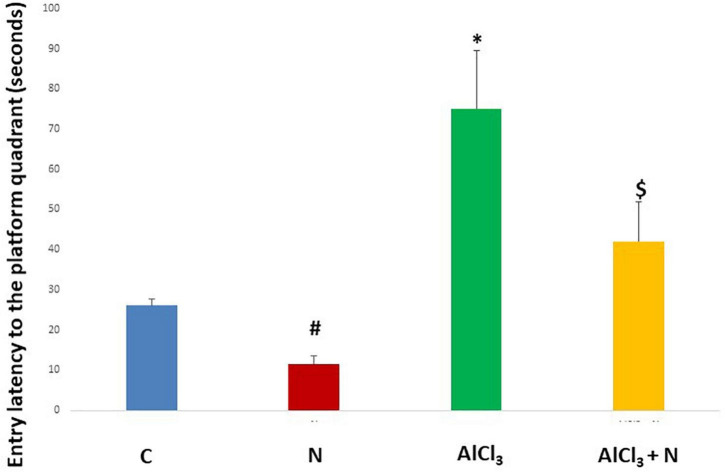
Entry latency in the Morris water maze test in the studied groups. *n* = 6 in each group. Data were prescribed as mean ± *SD*. The intergroup variation was conducted by Games-Howell *post-hoc* test. *p < 0.001 compared with C group. ^#^*p* < 0.001 compared with AlCl_3._^$^*p* < 0.001 compared with AlCl_3_.

During initial training periods, there were no significant differences among rats in the different studied groups regarding the time required to reach the platform (escape latency) (*p* = 0.3) or the time spent on the rod (grip period) (*p* = 0.2) (ANOVA test) (Figures 1, 2). After that, AlCl_3_-treated rats spent less time on the rotarod (grip period) (*p* < 0.001) and more time to enter the platform quadrant (entry latency) (*p* < 0.001) when compared with AlCl_3_ + N group (Figures 3, 4).

### Evaluation results of oxidative stress and lipid peroxidation markers

Compared to C group, AlCl_3_ group showed a significant increase in hippocampus MDA, NO, and a significant drop in GSH levels (*p* < 0.001, *p* = 0.002, *p* < 0.001, respectively). Hippocampal MDA and NO levels significantly decreased after NAR administration, while the hippocampal GSH level was significantly increased (Table 2).

**TABLE 2:** Effects of naringin administration on MDA, NO, and GSH levels in hippocampal and cerebellar homogenates.

	Hippocampus				
	
	C	*N*	AlCl_3_	AlCl_3_ + *N*	*P*-value
MDA (nmol/g)	10.1 ± 1.58	9.5 ± 1.25^#^	18.8 ± 2.1*	15.2 ± 2.3^$^	*P* < 0.001**
GSH (μmol/g)	1.65 ± 0.35	1.79 ± 0.48^#^	0.84 ± 0.12*	1.11 ± 0.21^$^	*P* < 0.001**
NO (μmol/g)	1.31 ± 0.47	1.25 ± 0.38^#^	2.37 ± 0.58*	1.98 ± 0.88^$^	*P* = 0.002**

	**Cerebellum**				

MDA (nmol/g)	15.5 ± 6.18	16.1 ± 2.48^#^	29.3 ± 6.85*	16.1 ± 2.48^$^	*P* = 0.004**
GSH (μmol/g)	2.14 ± 0.38	1.97 ± 0.26	1.15 ± 0.58	1.97 ± 0.26	*P* = 0.01
NO (μmol/g)	1.97 ± 0.77	2.15 ± 0.81^#^	3.41 ± 0.78*	2.15 ± 0.81^$^	*P* = 0.001**

*n* = 6 in each group. Data were prescribed as mean ± SD.

**Indicates significance among the studied groups indicated by ANOVA test. The intergroup variation was conducted by Games-Howell *post-hoc* test.

*Significance compared with C group.

^#^Significance compared with AlCl_3._

^$^Significance compared with AlCl_3_.

Furthermore, compared to C group, AlCl_3_ group generated a significant increase in cerebellar MDA, NO, and a significant decrease in GSH levels. When NAR and AlCl_3_ were administered together, the levels of MDA and NO decreased (*p* = 0.004, *p* < 0.001), respectively and GSH level increased significantly in the cerebellum (*p* = 0.01) than they were in AlCl_3_ group (Table 2).

### The effects of naringin intake on hippocampal histological architecture

**FIGURE 5:**
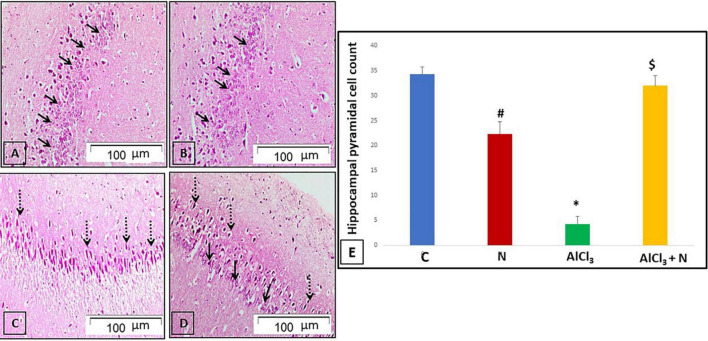
H & E-stained hippocampal sections revealing the cornu amonnis region 1 (CA1): (**A**,**B)** C and N groups, respectively showed its pyramidal cell layer formed of small pyramidal neurons with large vesicular nuclei and prominent nucleoli (arrows). **(C)** AlCl_3_ group showed that most of the pyramidal neurons appeared to be deeply stained with pyknotic nuclei (dotted arrows). **(D)** The group treated with both AlCl_3_ and *N* showed that most of pyramidal neurons appeared normal with large vesicular nuclei and prominent nucleoli (arrows). However, some pyramidal neurons appeared deeply stained with pyknotic nuclei (dotted arrows) (H & E, × 200). **(E)** Pyramidal cell count of all studied groups. *n* = 6 in each group. Data were prescribed as mean ± *SD*. The intergroup variation was conducted by Games-Howell *post-hoc* test. *p < 0.001 compared with C group. ^#^*p* = 0.004 compared with AlCl_3._^$^*p* < 0.001 compared with AlCl_3_.

Pathological examination of hematoxylin and eosin-stained hippocampal sections was performed to verify the neurochemical findings. Sections of C and N groups showed the pyramidal cell layer, which is composed of tiny pyramidal neurons with large vesicular nuclei and visible nucleoli, as well as the typical architecture of the hippocampal tissue (Figures 5A,B). Most pyramidal neurons in AlCl_3_ group appeared deeply stained with pyknotic nuclei (Figure 5C). On the other hand, most of pyramidal neurons in AlCl_3_ + *N* group appeared normal, with large vesicular nuclei and visible nucleoli. However, some pyramidal neurons had deeply stained pyknotic nuclei (Figure 5D).

### The effects of naringin intake on cerebellar histological architecture

**FIGURE 6:**
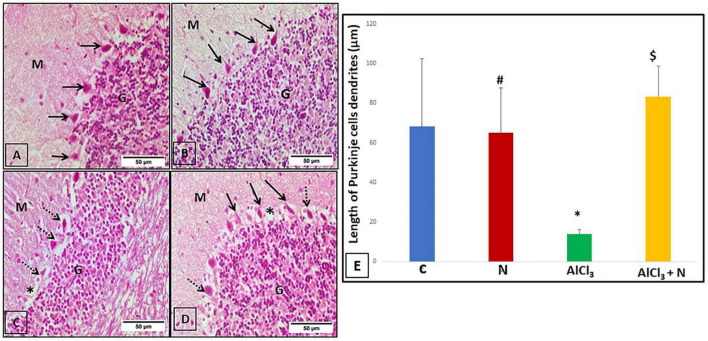
Cerebellar sections stained with H & E-stained cerebellar sections of the studied groups: **(A,B)** C and N groups, respectively, showed the three layers of the cerebellar cortex: molecular layer (M), Purkinje cell layer (arrows) and granular layer (G). Purkinje cells appeared pyriform in shape, with apical dendrite projecting upwards in the molecular layer. **(C)** The AlCl_3_ group showed normal appearance of both molecular (M) and granular (G) layers. Purkinje cells had a distorted shape with lost apical dendrites (arrows). **(D)** The AlCl_3_ + *N* group showed normal appearance of the molecular (M) and granular (G) layers. Most of the Purkinje cells appeared to be normal in shape having apical dendrites (arrows). However, some of the Purkinje cells had a distorted shape with the loss of their apical dendrites (dotted arrows). Also, there were empty spaces (*) indicating neuronal degeneration (H & E, × 400). **(E)** Length of the Purkinje cell dendrites of all studied groups. *n* = 6 in each group. Data were prescribed as mean ± *SD*. The intergroup variation was conducted by Games-Howell *post-hoc* test. *p < 0.001 compared with C group. ^#^*p* < 0.001 compared with AlCl_3._^$^*p* < 0.001 compared with AlCl_3_.

The three layers of the cerebellar cortex; the molecular layer, the Purkinje cell layer and the granular layer were seen in the cerebellar sections of C and N groups. Purkinje cells were pyriform in shape and had apical dendrites that extended upwards into the molecular layer (Figures 6A,B). The morphology of Purkinje cells was deformed and they lost their apical dendrites in AlCl_3_ group (Figure 6C). In AlCl_3_ + *N* group, most of Purkinje cells restored their pyriform shape and apical dendrites. Furthermore, there were empty spaces that indicated neuronal degeneration (Figure 6D).

### The effect of naringin on immunohistochemical detection of cerebellar microtubule-associated protein tau

**FIGURE 7:**
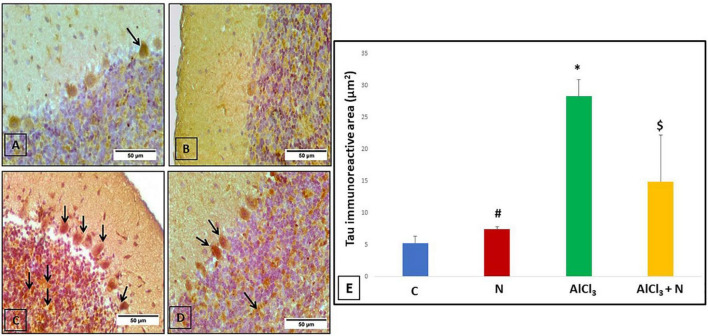
Immunohistochemical detection of cerebellar tau protein: **(A)** The control rat showed weak positive tau immunoreaction in a Purkinje cell (arrow). **(B)** The rat showed negative tau immunoreaction. **(C)** AlCl_3_ group showed a strong positive tau immunoreaction in most Purkinje and granule cells (arrows). **(D)** The rat treated with combined AlCl_3_ and N showed weak positive tau immunoreaction in some Purkinje and granule cells (arrows) (Tau × 400). **(E)** Area% of tau-positive immune reaction in cerebellar sections of all studied groups. *n* = 6 in each group. Data were prescribed as mean ± *SD*. The intergroup variation was conducted by Games-Howell *post-hoc* test. *p < 0.001 compared with C group. ^#^*p* < 0.001 compared with AlCl_3._^$^*p* < 0.001 compared with AlCl_3_.

C group showed weak negative tau reaction in the Purkinje cells (Figure 7A). Rats treated with NAR appeared as having a lower level of anti-tau antibodies than C group (Figure 7B). AlCl_3_ group revealed strong positive tau immunoreaction in the majority of Purkinje and granule cells (Figure 7C). Unlike AlCl_3_ group, some Purkinje and granule cells in AlCl_3_ + *N* group had weak positive tau immunoreaction (Figure 7D).

### The effect of naringin on immunohistochemical detection of cerebellar iNOS (oxidative stress marker)

**FIGURE 8:**
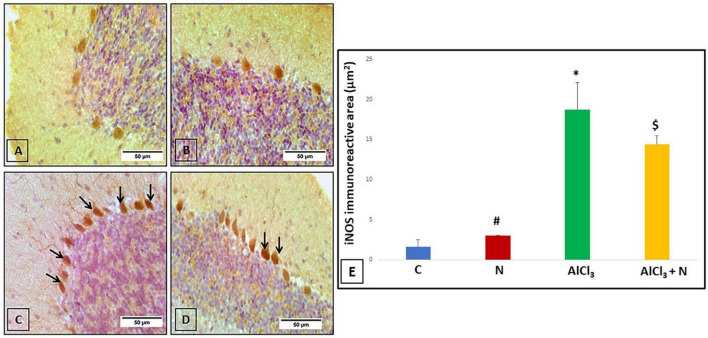
Immunohistochemical detection of cerebellar iNOS **(A,B)** C and N groups, respectively showed negative iNOS immunostaining. **(C)** AlCl_3_ group showed strong positive iNOS immunostaining in most Purkinje cells (arrows). **(D)** AlCl_3_ + *N* group showed weak positive immunostaining for iNOS in some Purkinje cells (arrows) (iNOS × 400). **(E)** Area% of iNOS positive immune reaction in cerebellar sections of all studied groups. *n* = 6 in each group. Data were prescribed as mean ± *SD*. The intergroup variation was conducted by Games-Howell *post-hoc* test. *p < 0.001 compared with C group. ^#^*p* < 0.001 compared with AlCl_3._^$^*p* < 0.001 compared with AlCl_3_.

Cerebellar sections were immunostained with iNOS to see if the neuroprotective effect of naringin was related to reduced oxidative stress or not. Both C and N groups showed negative iNOS reaction (Figures 8A,B). AlCl_3_ group showed that most of the Purkinje cells displayed strong positive iNOS immunostaining (Figure 8C). Unlike AlCl_3_ group, some Purkinje cells in AlCl_3_ + N group showed weak positive iNOS immunostaining (Figure 8D).

### The effect of naringin on immunohistochemical detection of cerebellar LC3 (autophagy marker)

**FIGURE 9:**
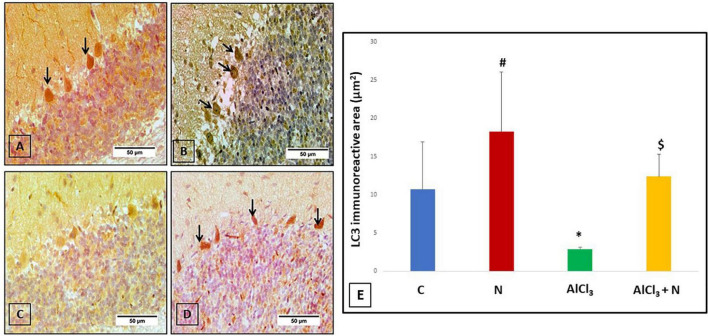
Immunohistochemical detection of cerebellar LC3: **(A,B)** C and N groups, respectively showed strong positive LC3 immunostaining in most of the Purkinje cells (arrows). **(C)** AlCl_3_ group showed strong negative LC3 immunostaining. **(D)** AlCl_3_ + *N* treated group showed weak positive immunostaining for LC3 in some Purkinje cells (arrows) (LC3 × 400). **(E)** Area% of LC3 positive immune reaction in cerebellar sections of all studied groups. *n* = 6 in each group. Data were prescribed as mean ± *SD*. The intergroup variation was conducted by Games-Howell *post-hoc* test. *p = 0.008 compared with C group. ^#^*p* < 0.001 compared with AlCl_3._^$^*p* < 0.001 compared with AlCl_3_.

To explore whether the neuroprotective effect of naringin was associated with autophagy, the changes of the autophagic vacuoles and the autophagic substrate were studied. Both C and N groups showed strong positive LC3 reaction (Figures 9A,B). Immunohistochemical analysis of AlCl_3_ group showed negative LC3 immunostaining (Figure 9C). Moreover, AlCl_3_ + *N* group showed positive LC3 reaction (Figure 9D).

### Quantitative measurement of hippocampal pyramidal cell count

Alcl_3_ group showed a significant reduction in the hippocampal pyramidal cell count compared to both C and N groups. Co-administration of NAR resulted in a significant increase in the pyramidal cell count as compared to AlCl_3_ group (*p* < 0.001) (Figure 5E).

### Quantitative measurement of the length of the dendrites of cerebellar Purkinje cells

The length of the dendrites of cerebellar Purkinje cells was significantly decreased in AlCl_3_ group as compared to both C and N groups. AlCl_3_ + *N* group showed a significant increase in dendritic length as compared to AlCl_3_ group (*p* < 0.001) (Figure 6E).

### Quantitative measurement of the percentage area of positive immunoreaction in cerebellar tissues

AlCl_3_ group showed a significant increase in the% area of tau immunoreaction as compared to both C and N groups (*p* < 0.001). NAR co-administration for 21 days showed a significant reduction in the% of area of positive tau reaction as compared to AlCl_3_ group (Figure 7E).

Regarding iNOS, AlCl_3_ group revealed a substantial increase in iNOS immunostaining as compared to both C and N groups (*p* < 0.001). NAR administration for 21 days demonstrated a significant decrease in iNOS immunostaining as compared to AlCl_3_ group (Figure 8E).

Regarding LC3, AlCl_3_ group revealed that the LC3 immunostaining was significantly reduced (*p* < 0.001) as compared to both C and N groups. Furthermore, LC3 immunostaining was significantly increased after NAR co-administration when compared with AlCl_3_ group (Figure 9E).

### The expression of p-Tau, Tau, iNOS and LC3-II/I proteins in the cerebellum using Western blot procedure

**FIGURE 10:**
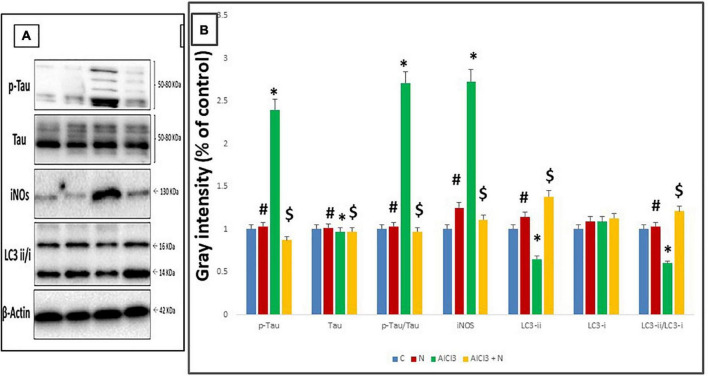
Western blot analysis. **(A)** Representative Western blot images of cerebellar tau, iNOS, and LC3 proteins. **(B)** Quantification of the proteins studied in cerebellar tissue lysates. *n* = 3 in each group. The control group is set to “1,” and data were prescribed as mean ± *SD*. The intergroup variation was conducted by Games-Howell *post-hoc* test. *Significance compared with C group. ^#^Significance compared with AlCl_3._^$^Significance compared with AlCl_3_.

Compared to C group, AlCl_3_ group showed a significant increase in p-Tau (Ser-396/404) and p-Tau/Tau and decreased total Tau levels. When NAR and AlCl_3_ were administered together, the expression of p-Tau (Ser-396/404) and p-Tau/Au decreased, while overall tau levels increased significantly when compared to AlCl_3_ group (Figure 10).

When NAR and AlCl_3_ were administered together, iNOS expression was significantly reduced as compared to AlCl_3_ group. AlCl_3_, on the other hand, significantly increased the expression of iNOS as compared to C group (*p* = 0.001) (Table 3).

**TABLE 3:** Effects of naringin administration on the expression of cerebellar LC3, tau and iNOS proteins by Western blot analysis.

	Control	Naringin	AlCl_3_	AlCl_3_ + Naringin	*P*-value
p-Tau	1.0 ± 0.23	1.03 ± 0.287^#^	2.40 ± 0.007*	0.870 ± 0.11^$^	0.002**
Tau	1.0 ± 0.079	1.01 ± 0.36^#^	0.973 ± 0.299*	0.974 ± 0.347^$^	0.04**
p-Tau/Tau	1.0 ± 0.30	1.03 ± 0.084^#^	2.71 ± 0.584*	0.965 ± 0.461^$^	0.004**
iNOS	1.0 ± 0.16	1.25 ± 0.12^#^	2.73 ± 0.135*	1.11 ± 0.09^$^	0.001**
LC3-ii	1.0 ± 0.056	1.14 ± 0.263^#^	0.652 ± 0.004*	1.38 ± 0.212^$^	0.001**
LC3-i	1.0 ± 0.12	1.09 ± 0.217	1.093 ± 0.17	1.132 ± 0.19	0.08
LC3-ii/LC3-i	1.0 ± 0.064	1.03 ± 0.03^#^	0.601 ± 0.089*	1.21 ± 0.17^$^	0.001**

*n* = 3 in each group. Data were prescribed as mean ± SD.

**Indicates significance among the studied groups detected by ANOVA test. The intergroup variation was conducted by Games-Howell *post-hoc* test.

*Significance compared with C group.

^#^Significance compared with AlCl_3._

^$^Significance compared with AlCl_3_.

Combined administration of NAR and AlCl_3_ resulted in a significantly higher expression of the LC3-i, LC3-ii, and LC3-ii/LC3-i ratio when compared with AlCl_3_ group. On the other hand, it significantly reduced the expression of LC3-ii and the LC3-ii/LC3-i ratio as compared to C group (*p* = 0.001) (Table 3).

**FIGURE 11:**
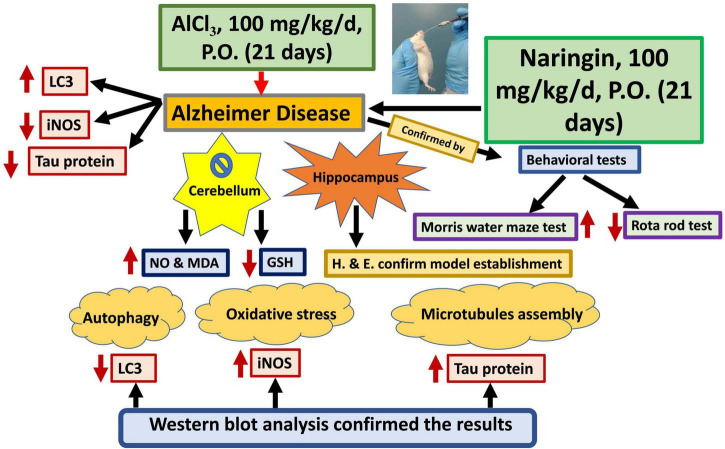
Graphical abstract. Naringin improves the cerebellar neurotoxicity induced by AlCl_3_ evaluated by decrease of the oxidative stress marker (in the form of decreasing the iNOS); decrease the microtubule assembly marker (Tau protein) and increase the autophagy marker (LC3). AlCl_3,_ Aluminum chloride; Inos, Inducible nitric oxide synthase; LC3, Light chain 3; GSH, Reduced glutathione; NO, Nitric oxide and MDA, Malondialdehyde.

A graphical abstract was designed to summarize all results of the current study (Figure 11).

## Discussion

The present study was designed to investigate the cerebellar changes in AlCl_3_ rat model of AD focusing on behavioral, neurochemical, immunohistochemical, and molecular aspects. Furthermore, the neuroprotective role of naringin against AD-related cerebellar changes was estimated.

AD is accompanied by neuronal loss and structural changes (Xiaoguang et al., 2018). Short-term memory impairment is the first clinical sign of AD due to hippocampal neuronal degeneration (Lakshmi et al., 2015). Al is believed to play a role in the development of AD due to its easy entry and accumulation in the central nervous system (Sun et al., 2009). Understanding the pathophysiology in neurodegenerative and neuropsychiatric diseases, particularly AD, has gained considerable interest in this area of research (Jacobs et al., 2018). Um et al. (2022) reviewed an already considerable work on cerebellar neuropathology, structural organization, and functional neuroimaging studies in AD.

According to the findings of the present study, administration of AlCl_3_ caused a significant decline in learning ability and spatial memory, assessed by the Morris water Maze test. Compared to AlCl_3_ group, AlCl_3_ + *N* group demonstrated a significantly shorter time (*p* = 0.001) to reach the platform in the Morris water maze in all four quadrants. Also, NAR co-administration improved the retention of memory evaluated by the time needed by rats to enter the platform quadrant. Lakshmi et al. (2015) declared that intracerebral treatment of AlCl_3_ resulted in learning deficits in rabbits in the Morris water maze test, which was consistent with our findings. This phenomenon could be attributed to the ability of Al to interfere with downstream effector molecules, such as cyclic GMP, involved in long-term memory (Canales et al., 2001). This disturbance could then clarify the memory impairment and neurobehavioral deficits detected. Also, Yang et al. (2014) supported the results of the current study as they mentioned that naringenin (naringin metabolite) can easily cross the blood brain barrier and improve spatial learning and memory in a rat model of AD by regulating the PI3K/AKT/GSK-3 pathway and reducing tau hyperphosphorylation. In addition, naringin improves memory deficits in experimental models of AD by attenuating mitochondrial dysfunction (Sachdeva et al., 2014).

In addition, the current study revealed that there was a significant decrease in the motor abilities and muscle coordination measured by the Rotarod test after AlCl_3_ administration. According to Wagner et al. (2019), sporadic AD is characterized by cerebellar atrophy, and cerebellar damage is the cause of motor impairment.

Furthermore, these results were in line with those of Chen C. et al. (2019) who found that daily intragastric administration of naringenin for 7 days attenuated the decrease in the time rats remained on the rotarod. In addition, Meng et al. (2021) found that naringin can significantly improve cognitive, learning, and memory dysfunction in mice with hydrocortisone memory impairment. They concluded that naringin exerted these neuroprotective effects through a variety of mechanisms, including amyloid-β metabolism, tau protein hyperphosphorylation, the acetylcholinergic system, the glutamate receptor system, oxidative stress, and cell apoptosis. Also, naringin is a potent antioxidant which is rapidly absorbed into the blood from the intestinal tract and is then further redistributed to other organs, including the brain (Wang et al., 2021). Feng et al. (2018) excluded that naringin can cross the blood brain barrier. Thus, it can protect the brain tissue and modulate brain chemistry.

The current study demonstrated that MDA and NO levels increased significantly as a result of AlCl_3_-induced AD. According to Liaquat et al. (2019), prolonged exposure to AlCl_3_ can harm brain DNA, affect brain neurochemistry, and alter antioxidant enzyme activities. Additionally, aluminum toxicity results in marked oxidative stress by raising pro-oxidant iron levels in the brain and decreasing antioxidant enzyme functions (Praticò et al., 2002).

AlCl_3_ increases the production of free radicals, which eventually causes oxidative stress and neurotoxicity. The brain is particularly susceptible to oxidative stress, which results in toxicity when free radicals increase and the antioxidant status declines (Kumar and Gill, 2014). MDA accumulates as a result of ROS production. It damages and deteriorates membranes by lipid peroxidation (Busch and Binder, 2017). In the same line, current results demonstrated a significant drop in GSH levels in AlCl_3_ group. Glutathione in its reduced form is the most abundant intracellular antioxidant and is involved in the direct scavenging of free radicals or serving as a substrate for the glutathione peroxidase enzyme that catalyzes H_2_O_2_ detoxification. Catalase is also known to be a protective enzyme and functions for the detoxification of highly reactive free radicals (Mačičková et al., 2005).

In the present study, naringin restored reduced glutathione and decreased NO and MDA levels in rats treated with both AlCl_3_ and NAR. Liaquat et al. (2018) reported that NAR was found to be the most effective antioxidant of polyphenols. Additionally, it has estrogenic properties, which alter NO generation by activating estrogen receptors (Ghofrani et al., 2015). Furthermore, NAR antioxidant effects modulate oxidative stress and inflammatory responses in the adult brain. Its neuroprotective effects are also controlled by induction of neurotrophic factors and activation of anti-apoptotic pathways (Kim et al., 2016).

According to the current work, AlCl_3_ caused an increase in iNOS expression which was studied at immunohistochemical and molecular levels in the cerebellar tissue, both of which were alleviated by NAR administration. In agreement with these findings, Dou et al. (2013) showed that in many forms of inflammation, naringenin has also been demonstrated to suppress iNOS expression. Furthermore, in mice whose brains had been exposed to 1-methyl-4-phenyl-1, 2, 3, 6-tetrahydropyridine (MPTP), pretreatment with naringenin reduced the degree of iNOS expression (Sugumar et al., 2019).

Histopathological studies were performed on the rat hippocampus to assess the morphological changes that confirmed the establishment of AD. Hippocampal neuronal degeneration was detected in the form of dark stained nuclei and increased pyramidal cell degeneration with noticeable neuronal distortion. These findings were confirmed by quantitative measurement of the hippocampal pyramidal cell count. The count was significantly reduced in AlCl_3_ group as compared to AlCl_3_ + *N* group (*p* < 0.001). Chen et al. (2018) reported similar histological alterations like hippocampal neuronal degeneration as one of the main pathological hallmarks of AD. Furthermore, Haider et al. (2020) showed that pretreatment with naringin greatly inhibited AlCl_3_-caused Histological changes.

In the current study, Histological examination of cerebellar sections revealed that most of Purkinje cells in rats treated with AlCl_3_ had deformed shapes and lost their apical dendrites. These changes improved in AlCl_3_ + *N* group. In addition, AlCl_3_-treated rats had shorter dendritic length when compared with AlCl_3_ + *N* group (*p* < 0.001). These neuropathological and morphological alterations were in agreement with Baloyannis et al. (2000) who revealed reduced Purkinje cell densities and significant morphological alterations in AD, such as loss of distal dendritic segments, lower dendritic densities and fewer dendritic branches and spines. Furthermore, the pattern of neurodegeneration in the cerebellum in AD may be explained by the dissemination of neurotoxic chemicals through neuronal pathways that connect dispersed nodes to functional modules through self-propagation or prion-like mechanisms (Mavroudis et al., 2019).

In the current study, the cerebellum of rats treated with AlCl_3_ had significantly higher levels of expression of p-Tau and p-Tau/Tau and the levels decreased significantly after receiving NAR. In the normal brain, tau binds to microtubules to stabilize them and accelerate axonal transport (Kontaxi et al., 2017). However, tau is hyperphosphorylated in AD, which causes it to separate from microtubules and assemble in the paired helical filaments and dystrophic neurites (Spillantini and Goedert, 2013). These results were in agreement with Ballaed et al. (2011) who found that activation of phosphokinase glycogen synthase kinase-3 (GSK-3) contributes to tau hyperphosphorylation of tau and is linked to the suppression of the PI3K/AKT pathway. Interestingly, the administration of naringin improved AD by lowering tau hyperphosphorylation (Yang et al., 2014).

For evaluating autophagy, the current work studied the expression of the LC3-II/I protein in cerebellar tissues using Western blot method. Furthermore, cerebellar sections were immunostained with LC3. Immunohistochemical examination of the cerebellum of AlCl_3_-treated rats revealed strong negative immunostaining of LC3 which became strong positive on NAR intake. These results indicated that AlCl_3_-induced AD created a state of autophagic stress in the cerebellum. Keller et al. (2004) supported our findings when they explained that autophagic stress generally refers to a relatively sustained imbalance in which the rate of autophagosome formation exceeds the rate of its degradation. In addition, neurons are vulnerable to defective autophagy due to their special features like having fewer lysosomes in distal axons and the axonal autophagosomes must be transported to the cell body to be combined with lysosomes (Cheng et al., 2015).

The current study revealed that the combined administration of NAR and AlCl_3_ resulted in significant higher expression of the LC3-i, LC3-ii, and LC3-ii/LC3-i ratio as compared to AlCl_3_-treated rats. These findings were in agreement with Boland et al. (2018) who established AD model in mice and found that the brain exhibits failure of lysosomal proteolysis. Park and Chung (2019) confirmed that there was a strong relationship between defective autophagy and AD pathogenesis. In addition, Jeong et al. (2015) declared that NAR may have beneficial effects in preventing neuronal death through anti-autophagic stress and anti-neuroinflammation in the hippocampus *in vivo*.

## Conclusion

The present study highlights that naringin improves behavioral, neurochemical, immunohistochemical, and molecular parameters in the rat cerebellum of AlCl_3_-induced AD; these effects may be largely attributed to its antioxidant and autophagic regulatory properties.

## Limitations of the study

To fully understand how naringin affects oxidative and autophagic stress in various experimental circumstances, more research is necessary.

## Data availability statement

The raw data supporting the conclusions of this article will be made available by the authors, without undue reservation.

## Ethics statement

Following NIH and EU norms for animal care, this study received Institutional Research Board (IRB) approval from the Faculty of Medicine at Mansoura University (Code number: R.21.03.1280). At the Medical Experimental Research Center (MERC), where the experiment was conducted, the rats were housed under veterinary care. The number of animals used and animal discomfort were both minimized as far as possible.

## Author contributions

HH and ME designed the study. HH examined the hippocampal and cerebellar tissue specimens, interpreted the histological and immunohistochemical results, and performed and interpreted the morphometric studies. EA and EE established the study model. MRE and EH performed and interpreted the neurochemical and molecular results. ZA-Q interpreted the results of behavioral tests. EMEN, MA, KA-K, and ZA-Q shared project administration, funding acquisition, investigation, methodology, writing, reviewing, and editing. All authors contributed to the conception of the study, revised the manuscript, and approved the submitted manuscript.
